# Platelets in pediatric and neonatal sepsis: novel mediators of the inflammatory cascade

**DOI:** 10.1038/s41390-021-01715-z

**Published:** 2021-10-28

**Authors:** Daniel O’Reilly, Claire A. Murphy, Richard Drew, Afif El-Khuffash, Patricia B. Maguire, Fionnuala Ni Ainle, Naomi Mc Callion

**Affiliations:** 1 Department of Neonatology, Rotunda Hospital, Dublin, Ireland; 2 Conway-SPHERE Research Group, Conway Institute, University College Dublin, Dublin, Ireland; 3 Department of Paediatrics, Royal College of Surgeons in Ireland, Dubin, Ireland; 4 Clinical Innovation Unit, Rotunda Hospital, Dublin, Ireland; 5 Irish Meningitis and Sepsis Reference Laboratory, Children’s Health Ireland at Temple Street, Dublin, Ireland; 6 Department of Clinical Microbiology, Royal College of Surgeons in Ireland, Dublin, Ireland; 7 School of Biomolecular & Biomedical Science, University College Dublin, Dublin, Ireland; 8 Department of Haematology, Mater Misericordiae University Hospital, Dublin, Ireland; 9 Department of Haematology, Rotunda Hospital, Dublin, Ireland; 10 School of Medicine, University College Dublin, Dublin, Ireland

## Abstract

**Abstract:**

Sepsis, a dysregulated host response to infection, has been difficult to accurately define in children. Despite a higher incidence, especially in neonates, a non-specific clinical presentation alongside a lack of verified biomarkers has prevented a common understanding of this condition. Platelets, traditionally regarded as mediators of haemostasis and thrombosis, are increasingly associated with functions in the immune system with involvement across the spectrum of innate and adaptive immunity. The large number of circulating platelets (approx. 150,000 cells per microlitre) mean they outnumber traditional immune cells and are often the first to encounter a pathogen at a site of injury. There are also well-described physiological differences between platelets in children and adults. The purpose of this review is to place into context the platelet and its role in immunology and examine the evidence where available for its role as an immune cell in childhood sepsis. It will examine how the platelet interacts with both humoral and cellular components of the immune system and finally discuss the role the platelet proteome, releasate and extracellular vesicles may play in childhood sepsis. This review also examines how platelet transfusions may interfere with the complex relationships between immune cells in infection.

**Impact:**

- Platelets are increasingly being recognised as important “first responders” to immune threats.
- Differences in adult and paediatric platelets may contribute to differing immune response to infections.
- Adult platelet transfusions may affect infant immune responses to inflammatory/infectious stimuli.

## Introduction

Sepsis, a dysregulated host response to infection, has been recognised as a global threat to both adults and children by the United Nations World Health assembly and a priority for the World Health Organisation to address.^1^ Despite this, accurately defining sepsis in children is fraught with difficulty. It has been challenging to generate paediatric definitions that adequately combine the unique paediatric pathophysiological response with adult Sepsis 3 criteria, and attempts to do so have yet to be validated outside of an intensive care setting.^1^ In neonates this difficulty is compounded by a high incidence of culture-proven sepsis with non-specific clinical presentations.^2^ Globally there is an estimated 3 million cases of neonatal sepsis and 1.2 million cases of paediatric sepsis a year.^3^ This leads to a situation where many paediatricians and neonatologists treat suspected sepsis empirically, as delayed diagnosis is associated with worse outcomes.^4^ Despite decades of interest, no single or set of validated biomarkers have been developed which accurately diagnose sepsis over a significant bacterial infection in the absence of a dysregulated host response.^5^

Anucleate platelets, derived from megakaryocytes, traditionally function in haemostasis and thrombosis. However an appreciation of the wider role of platelets as “first responders” in host defence has emerged recently with important roles in wound healing, innate and adaptive immunity described.^6^ Normal platelet production is driven by the production of thrombopoietic factors which stimulate megakaryocytopoiesis by increasing the number of megakaryocyte progenitors. These then differentiate, mature and ultimately release platelets into the bloodstream.^7^

The interest in platelets as mediators of an inflammatory cascade has an impact in how we view both neonatal and paediatric sepsis. Neonatal platelets have previously been shown to have a different functional and transcriptomic profile to their adult counterparts, suggesting their response to infection is likely to differ.^8–10^ How long this platelet “hyporesponsiveness” persists into childhood is not yet fully elucidated with almost adult response to agonists demonstrated at 14 days of life but with documented differences up until 15 years of age.^11,12^ Additionally, how this interacts with other parts of the immune system, which also demonstrate age-specific responses, is poorly described.

The purpose of this review is to place into context the role of paediatric platelets in the development of fulminant sepsis. To approach this we will examine what features of the platelet may contribute to its role outside of thrombosis, investigate the use of traditional platelet indices in paediatric sepsis and infection, and explore emerging molecular mechanisms underpinning platelet response to infection and why these might differ in both the neonatal period and childhood. Finally we will look at how this understanding could contribute to the development of a universal definition of sepsis through novel platelet derived extracellular vesicles (EVs) and proteomics, and examine gaps in the current literature which need to be addressed in order to fully understand platelets as key immune regulators in sepsis.

## Platelet indices and paediatric sepsis

**Fig. 1:**
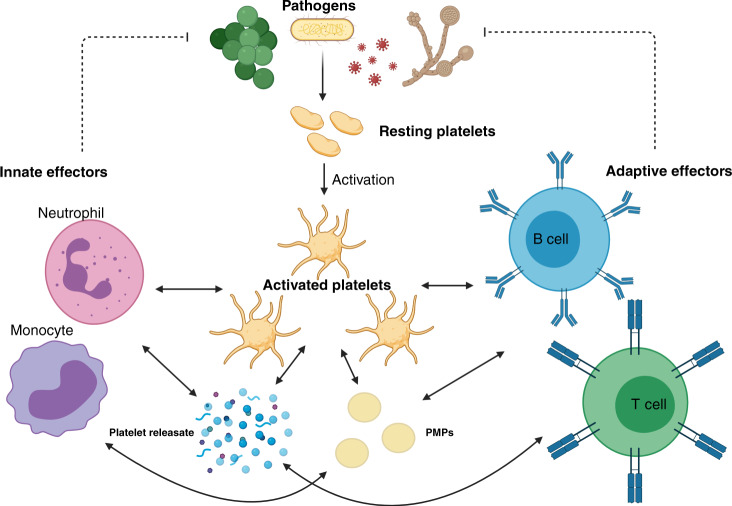
Visual abstract showing crosstalk between activated platelets, the innate and adaptive immune systems to coordinate an initial response against fungal, viral and bacterial pathogens. Solid arrows represent cell communication while broken arrows demonstrate target of immune response. Created with biorender.com.

Several platelet indices have been utilised as non-specific means of both prognostication and diagnosis in paediatric sepsis.^13,14^ Thrombocytopenia is a well-recognised component of both neonatal sepsis and severe paediatric sepsis.^13,15–17^ Thrombocytosis is a rarer finding in the septic child, and may represent a rebound phenomenon following a period of relative thrombocytopenia.^18^ Metrics such as platelet distribution width (PDW), which describes the range in platelet size, and mean platelet volume (MPV), the measure of average platelet size, have been used to predict outcomes in paediatric and neonatal sepsis.^13,19^ Changes in these parameters during sepsis offer a crude insight into the multiple roles played by platelets in sepsis pathophysiology (Fig. 1).

## Development of thrombocytopenia in paediatric and neonatal sepsis

A low platelet count is described in a large number of PICU and NICU admissions with sepsis^16,20^ A number of pathological mechanisms are thought to contribute to this process. One of the best understood is the development of a consumptive coagulopathy, i.e., disseminated intravascular coagulation (DIC), where platelets are inappropriately activated due to widespread vascular inflammation leading to the development of intravascular microthrombotic events throughout the systemic circulation. These consume both platelets and coagulation factors, leading paradoxically to increased bleeding and clotting. These events likely occur on a spectrum even in the absence of fulminant DIC, leading to a reduction of platelet count without necessarily associated signs of inappropriate bleeding or clotting.^21^

The bone marrow as the site of thrombopoiesis becomes both exhausted and suppressed as part of the dysregulated host response in sepsis.^22^ Septic neonates produce an excess of thrombopoietin (TPO), and the eventual development of thrombocytopenia may represent an “exhaustion” of marrow resident megakaryocytes and their precursors rather than a depression.^23,24^ This is particularly important as the neonatal megakaryocyte precursors, while responsive to TPO, tend to increase in number but not in size in response to stimulation. MPV tends to increase in response to TPO stimulation as larger, more immature platelets are released, leading to more rapid depletion of the rapidly differentiated, small, megakaryocytes.^7^ The association of increased MPV and thrombocytopenia with mortality in one study of sepsis in a PICU setting suggested that in septic children, bone marrow exhaustion may represent an almost preterminal event.^14^

Another measure of megakaryocytopoiesis which has been examined in neonatal sepsis and necrotising enterocolitis is immature platelet fraction (IPF). This is a measure of larger and more fluorescent platelets suggesting immaturity. IPF was shown to be higher prior to diagnosis of NEC or sepsis compared to previously reported healthy neonates and then declined reaching a nadir 3–5 days following diagnosis. This again supports a process of initial stimulation, possibly before the development of clinical signs, followed by bone marrow exhaustion.^25^

Developmental factors in paediatric patients may cloud this issue, as factors such as MPV and PDW may be partly age-dependent (see Table 1).^26,27^ A morphological transition from relatively small uniform megakaryocytes to a heterogeneous mixture of sizes commences from around 24 months with a typical “adult” appearance not achieved until 4 years of age.^28^ The functional consequences of this in the context of neonatal, infant and pre-school age children who are septic is as of yet undescribed.

**Table 1:** A comparative table of the features of platelets in health and sepsis between neonates, children and adults.

	Neonate	Child	Adult
Platelet count in healthy subjects^134^	150–400 × 10^9^/L: platelet numbers reach adult levels by end of the second trimester	150–400 × 10^9^/L Trends higher in infancy and decreases towards adolescence	150–400 × 10^9^/L
MPV^135^	Reduced in the very preterm cohort	Increases until adolescence, lower end of adult reference range	7.5–12 fL
PDW^27^	Higher in the preterm cohort	No specific reference range, similar to adulthood in health	10–17.9%
Megakaryocytes^28^	Small	Pattern of larger and smaller cells starting at 2 years old, adult distribution approached at 4 years old	Larger cells
Response to TPO^136^	Increase in number	Nil specific studies likely both neonatal and adult populations present at least until 4 years old	Increase in number and size
Surface glycoprotein expression^135,137^	Reduced levels of GpIIb/IIIa in all neonates and a reduction in Gp IIIa in preterm neonates	Reduced GpIIb/IIIa expression documented until 15 years old GpIb similar to adults	No difference in surface expression of GpIb
Response to agonists^138–140^	Reduced platelet activation in response to TRAP, thrombin, ADP/epinephrine and thromboxane A2 reaching near adult levels at 14 days	Near adult responses	Normal adult platelet activation profile
In sepsis			
	NICU	PICU	Adult ICU
Incidence of thrombocytopenia^20,141,142^	~49% of septic patients	Up to 25% of septic patients admitted to PICU	43–70% of sepsis admitted to ICU
Changes in other platelet parameters^14,25,143^	Increased MPV High IPF which then decreases	Increased MPV	Increased MPV High PDW

Bone marrow suppression due to the stimulation of Toll-like receptor 4 (TLR 4) and downstream signalling through Myeloid differentiation primary response 88 (Myd88) and TIR domain inducing adaptor inducing interferon β (TRIF) is well-described.^22,29^ Experimental models using lipopolysaccharide (LPS) or animal models of sepsis have permitted elucidation of this pathway in haematopoietic stem cells, megakaryocytes and platelets.^30^ While the latter are primed by TLR 4 stimulation and thus consumed in clot formation, controversy exists over possible further roles for TLR 4 in platelet immune function. Megakaryocytes and their progenitors are suppressed by Myd88/TRIF.^22^ Granulopoiesis, or the production of neutrophils, is favoured in the septic bone marrow by TLR 4/TRIF signalling to produce granulocyte colony-stimulating factor (G-CSF), leading to depletion of megakaryocytes and their precursors.^31^ In severe sepsis this leads to unchecked proliferation of monocytes from granulocyte/macrophage progenitor cells. These consume common megakaryocyte-erythroid precursors, erythrocytes and platelets in a phenomenon known as haemophagocytosis, which further exacerbates septic thrombocytopenia.^32,33^

## Platelets and pathogens

Platelets interact directly with pathogen-associated molecular patterns (PAMPs). LPS, found in Gram-negative organisms, activates a number of Toll-like receptors (TLRs) including TLR 4, TLR 6 and TLR 9 (ref. ^34^). This leads to sequestration and aggregation of platelets in the microvasculature of the liver and lungs.^35^ Interactions between stimulated platelets and neutrophils lead to neutrophil extracellular traps (NETs) formation, which capture circulating bacteria but also promote platelet aggregation, further reducing the circulating platelet count in sepsis.^36^

A number of pathogens have been shown to interact with platelets directly. Bacteria such as *Streptococcus pyogenes* and *Staphylococcus aureus* have specific virulence factors that target platelets and impair their function.^37–40^ Fungal interactions with platelets have been described given the long association between invasive fungal infection and thrombocytopenia.^41^ *Candida* species appear to inhibit platelet responses while *Aspergillus* spp. are potent activators of platelets which may contribute to the characteristic necrosis accompanying these infections.^41–43^ These virulence factors may have evolved as a means of pathogen circumvention of platelet facilitation of the immune response.

## Platelets as associate innate immune cells

**Fig. 2:**
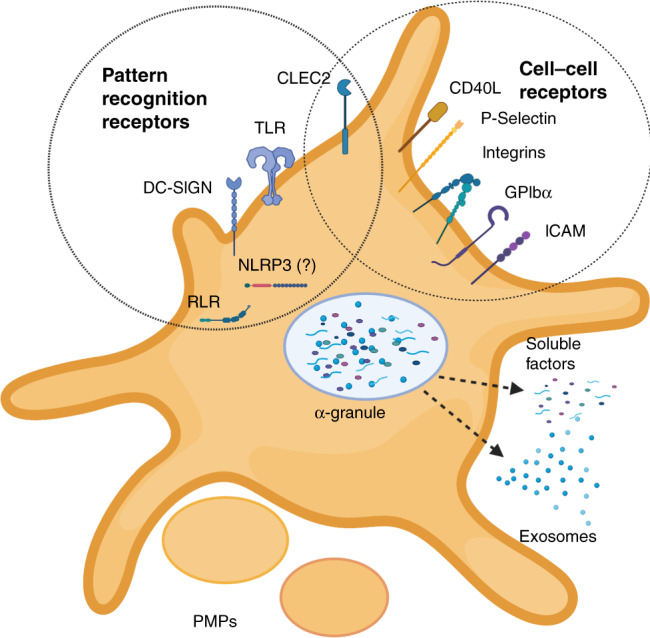
Activated platelet displaying pathogen recognition receptors (PRRs) alongside cell–cell receptors. Activated platelet is releasing soluble factors (cytokines, chemokines, lipid mediators, etc.) and exosomes through degranulation. Platelet also sheds larger platelet microparticles (PMPs) (created with biorender.com).

Platelets possess a suite of pattern recognition receptors (PRRs) including members of the TLR family, C-type lectin receptors (CLRs), NOD or nucleoside oligomerisation domain-like receptors (NLRs), and retinoic acid-inducible gene I (RIG-I)-like receptors (RLRs) (Fig. 2). While these contribute to sequestration and consumption of platelets in the septic child and neonate, their function is to allow platelets to lead the initial response to noxious stimuli through rapid degranulation and release of directly antimicrobial effectors.^44,45^ Platelets also facilitate the recruitment of other innate immune effectors such as granulocytes, monocytes and innate lymphoid cells (ILCs) including natural killer (NK) cells.^46^ These functions act in concert in both adult and paediatric sepsis, but important differences in these cell lines in children contribute to an altered host response.

## PRRs on platelets: signalling and roles

TLRs are among the best described signalling pathways as they are both highly conserved and widespread across different cell types.^47^ Nevertheless, the initial discovery of TLRs on the surface of platelets was unexpected as traditional TLR signalling pathways terminate in nuclear receptors leading to transcriptional alterations, e.g., nuclear factor κB (NFκB), which should not be possible in an anucleate cell.^34^ In platelets, the mechanism of action appears to be driven by altered transcription of pre-packaged mRNA. This is likely driven by signals from endogenous spliceosome components.^48^ Similar signalling pathways are utilised by other PRR classes.

Potential barriers to TLR signalling in platelets include the absence of co-receptors on the platelet surface. The co-receptor for TLR 4, CD14, is not present on platelets, but their response to a TLR 4-driven stimulus is potentiated in the presence of soluble CD14, an acute phase reactant increased in the presence of infection and inflammation.^34,49^

Preterm neonates have lower expression of TLR 4 and MyD88 on monocytes, and reduced response to LPS and lipoteichoic acid in vitro, which may contribute to the high rate of severe bacterial infections and sepsis in this cohort.^50^ There is a paucity of data on similar expression in other myeloid cell lineages including preterm and neonatal platelets.

Among the best known NLR receptors is NLRP3 (NOD-, LRR-, Pyrin-domain-containing protein 3). This cytosolic sensor identifies a wide range of signals including PAMPs and signs of cellular damage, referred to as danger-associated molecular patterns (DAMPs). It then forms the NLRP3 inflammasome, a complex of the NLRP3, ASC (apoptosis-associated speck like protein containing a CARD) and pro caspase 1, which drives production of IL-1β and IL-18 and pyroptosis.^51^ While several papers have demonstrated the presence of NLRP3 in platelets using flow cytometry techniques, with colocalization occurring in activated platelets, other transcriptomic methods failed to show evidence of the NLRP3 inflammasome components and argued against its presence within platelets.^52–55^ The study using transcriptomic methods noted that platelet co-culture with macrophage, monocytes and neutrophils appeared to drive increased NLRP3 activation in those cell lines, acting as a potentiator of the innate inflammatory response.^55^ Another study characterised platelet and NLRP3 activation in a rat model of polymicrobial sepsis (caecal ligation and puncture), showing that greater platelet NLRP3 activation was associated with end organ dysfunction.^52^ In neonatal studies, NLRP3 has been studied predominantly in the context of hypoxic ischaemic encephalopathy and chronic lung disease rather than sepsis and in serum as opposed to in platelets.^56,57^ Other inflammasomes have received even less attention.^58^ The presence of NLRP3 in platelets is likely to be a topic of for future debate. Age-dependent functional differences particularly in septic patients remain poorly described.

The presence of C-type Lectin-like receptors (CLRs) is well established on the surface of platelets, with numerous studies describing C-Type lectin Receptor 2 (CLEC 2) and dendritic cell (DC)-specific intracellular adhesion molecule 3 grabbing non-integrin (DC-SIGN) and their functions.^59,60^ CLEC 2 interacts with podoplanin, expressed by activated monocytes. A study which characterised *Salmonella typhi* infection in a mouse model reported increased thrombosis as a consequence of this interaction particularly in the liver using a mixture of immunohistology and flow cytometry,^61^ whereas another (using two separate mouse models of sepsis) showed an immunoregulatory role with reduction in inflammatory monocyte activity without thrombosis.^62^ It is unclear whether these differences reflect an organism-specific interaction, a context-dependent reaction according to other interacting cell types, or that a prothrombotic and antinflammatory effect occur simultaneously or at different points in the course of human sepsis. CLEC 2 has been examined alongside glycoprotein VI (GPVI), which signals through the same intracellular pathway in both preterm and term neonates, where agonist rhodocytin was used to activate these receptors. Expression of activated integrin αIIbβ3 was used to characterise responsiveness of platelets. This study showed both reduced expression of CLEC 2, GPVI and αIIbβ3 at transcriptional level and diminished response.^63^ It was suggested that this might represent a more generalised reduction in responsiveness to immunotyrosine-based activation motif (ITAM)-containing receptors. The GPVI-spleen tyrosine kinase (SYK) signalling cascade has been shown recently to be downregulated in critically ill adult patients with sepsis, hinting towards a key role in sepsis pathophysiology.^64^ Given the known hyporesponsiveness of TLR 4 in monocytes from this age group, and the fact that SYK is involved in both pathways, it is tempting to hypothesise that this represents a key intracellular step in the relative immunodeficiency in neonates.^65^

DC-SIGN is a CLR which binds to mannose or lewis x carbohydrate structures in order to recognise a wide range of pathogens. It has been most studied for its role in immune evasion by HIV and TB.^66^ Platelets have been shown to express DC SIGN, and its role is widely understood in the context of viral host defence^59,67^; however, there is limited information available on its role in bacterial infections, or on any differences between paediatric and adult platelet expression or signalling. Similarly RLRs research has predominately examined viral infection in megakaryocytes, without investigation of any maturational changes in different age groups.^68^

In summary, platelets express a wide variety of PRRs allowing recognition of PAMPs and DAMPs, which are associated with a range of immunological insults including sepsis (Fig. 2). While early efforts have been made to describe differences between these pathways in adults and neonates, there remains a gap in the knowledge regarding how and when these pathways mature, and how maturation affects the differing pathophysiology of sepsis from childhood to adulthood.

## Platelet interactions with other innate immune effector cells

Platelets also interact with other innate effector cells through direct contact and through the release of a myriad of paracrine and autocrine signalling molecules through their platelet releasate.^69–71^ Interactions between platelets and other immune effector cells are therefore complex and context dependent, with many gaps in the current literature particularly with respect to paediatric and neonatal disease processes.

The neutrophil–platelet interaction is among the best understood, due to the phenomenon of NETs and their prominent role in the innate immune response.^72^ Previous reviews have described the plethora of signalling pathways resulting in NETosis, the release of a DNA and protein web which captures pathogens and prevents their dissemination.^72^ TLR 4 has been shown to play a key role in the initial platelet–neutrophil interaction by activating both cell types when stimulated.^36^ Activated platelets express P-selectin interacts with P-selectin glycoprotein ligand 1 (PSGL-1), expressed on the surface of neutrophils and other leucocytes. This facilitates the interaction of high mobility group protein B1 (HMGB1) from platelets with the receptor for advanced glycosylation end-products (RAGE) by spatial approximation of the two cell types. An intracellular signalling cascade within neutrophils is then triggered, which produces reactive oxygen species (ROS) and promotes neutrophil elastase translocation to the nucleus, where it digests chromatin. This in turn results in the release of free DNA for incorporation into NETs alongside granular antibacterial proteins. Intracellular regulators then produce either lytic NETosis, through death of the neutrophil, or the more rapid non-lytic NETosis.^72^ Animal models have shown this to be a key component of the inflammatory response in sepsis.^73^ Evidence of NETosis has also been demonstrated in clinical studies examining meningococcal sepsis in a PICU setting.^74^ While NET formation is a key point in neutrophil–platelet interactions it is important to note that while platelets are important in augmenting NET formation they are not necessary for this to occur.^72^

Term and preterm infants not only exhibit hyporeactive platelets and reduced PRR signalling (particularly TLR 4), but reduced NETosis has also been demonstrated in these patients in some studies, which could contribute to the high rate of sepsis in the newborn period.^75^ Reduction of NETosis in the immediate newborn period has been ascribed to a series of related peptides released from the placenta including neonatal NET inhibitory factor.^76^ However, conversely, NET production was increased in an “infant” mouse model of sepsis and associated with worse organ injury and greater pro-inflammatory cytokine production.^77^ Whether this represents a difference between species, or species-specific immune development, is difficult to ascertain from existing literature. While NETs are hypothesised to reduce the spread of infection they are also associated with the development of DIC and microvascular damage.^73^ Determining the contribution of platelet–neutrophil interactions to the neutrophil hypofunction of the neonatal period has yet to be described. Additionally, the timing of when a more “mature” effector neutrophil function develops has yet to be elucidated.

Beyond the formation of NETs, the neutrophil–platelet interaction is characterised by both chemical and biomechanical crosstalk. Platelets can slowly migrate and “bundle” invading bacteria so they are more localised and vulnerable to neutrophil phagocytosis/NET formation.^78^ Similarly, neutrophils “scan” for activated platelets to indicate where injury, inflammation or infection is occurring.^79^ Relevant interactions occur not only through the P-Selectin–PSGL-1 complex but through a wide range of integrins. These include LFA 1 (CD11a/CD18), which interacts with ICAM 1 expressed on the platelet surface, and Mac 1 (CD11b/CD18), which binds GPIb.^80,81^ Platelets themselves tend to be pushed towards vessel walls due to their relatively small size compared with erythrocytes.^82^ This gives them ample opportunity to activate and interact with endothelial cells, increasing the expression/translocation of integrins from weibel-palade bodies.^83^ These mechanical interactions allow platelets to facilitate and enhance the neutrophil functions of adhesion, rolling and transmigration through the endothelial wall to sites of inflammation and infection. Neonatal neutrophils exhibit reduced expression of Mac 1, which likely impairs their ability to effectively localise to sites of inflammation/infection.^84–86^ This may explain the neonatal increased susceptibility to sepsis, as they mount a relatively weak innate response to localised infections.

A number of major components of platelet α granules also facilitate neutrophil activation and chemotaxis. Notably, the chemokines platelet factor 4 (PF4/CXCL4), RANTES (CCL5) and neutrophil-activating peptide-2 (NAP-2/CXCL7) are involved in endothelial adhesion, chemotaxis and transmigration.^80^ These signals are in turn regulated by chemical signalling from the neutrophil itself with cathepsin G, a neutrophil-derived serine protease, potentiating these reactions. Signals including phospholipids (such as platelet activating factor/PAF), nitric oxide, ROS and other cell surface proteins are contribute to this complex interplay between platelets, neutrophils and the endothelium.^80,81^ These interactions are both site-specific and context dependent. For example, acidosis, a common finding in the perinatal period and neonatal sepsis, deviates platelets away from traditional haemostatic and thrombotic roles and towards increased immunological activity.^87^ How these factors contribute to the neonatal response to sepsis is not yet known, and there is even less information available about how maturation of this cellular interplay contributes to the pathophysiology of sepsis in later infancy and early childhood.

More information has been elucidated about platelet–neutrophil interactions than other models of traditional immune cell and platelet crosstalk, but there is substantial evidence that similar communication mechanisms exist with other immune cells. Type 2 ILCs have been shown to express PSGL-1 and are involved in polymicrobial sepsis responses.^88,89^ A number of interactions have been also noted between NK cells and platelets in the oncological states.^90^ Although both cell types play key roles in sepsis, and exhibit maturational, age-dependent changes from childhood to adulthood, no research looking at the relationship between NK cells and platelets in childhood sepsis was found.^91,92^

Platelets also have numerous interactions with specialised antigen-presenting cells, which act as a bridge between the innate and adaptive immune systems. Phagocytosis of platelet precursors and platelets in bone marrow is a feature of sepsis and other inflammatory disease (including the sepsis mimic haemophagocytic lymphohistocytosis/HLH).^33,93^ However, a more nuanced relationship between these cell types also exists. Monocyte–platelet complexes are formed using many of the same receptors utilised in neutrophil–platelet interactions, with P-selectin on activated platelets and PSGL-1 on monocytes being key initiators of this reaction. These are then strengthened with multiple enforcing reactions through HMGB-1-RAGE, CLEC 2-Podoplanin and glycoprotein Ib (GPIb)-CD11b interactions.^94^ The resulting complex function is situation-dependent, and is most commonly described with a pro-inflammatory profile, but with substantial evidence of immune regulatory/anti-inflammatory profiles in certain situations.^94,95^ When pro-inflammatory, it leads to an increase in pro-inflammatory cytokines and chemokines, tumour necrosis factor α (TNF-α), IL-1β, monocyte chemoattractant protein 1 (MCP-1/CCL2) and IL-8 among others.^94^ This phenotype has predominated in the sepsis literature to date, with increased platelet monocyte aggregates associated with in Gram positive as opposed to Gram-negative bacteraemia, and a similar increase in aggregates is linked to a higher mortality in adult sepsis.^94^ No studies have attempted to replicate these findings in children or neonates, despite known difference in the monocytic response to sepsis in children and neonates.^96–98^

The P-Selectin–PSGL-1 interaction acts as a key checkpoint in the maturation of monocytes into DCs.^99^ Activated platelets also express CD40L, an important stimulator of DC function which is required for an effective adaptive immune response.^100,101^ Kupffer cells, the resident macrophages of the liver, “capture” circulating platelets in liver sinusoids and use them to encase bacteria using the GPIb–CD11b interaction.^102^ This contributes to the propensity for clot formation in the liver in the context of sepsis.

Platelets also influence antigen presentation through their interaction with the complement cascade. This essential component of humoral immunity acts by separate pathways to facilitate direct lysis of threats, through the formation of the membrane attack complex or by “tagging” threats for phagocytosis (opsonisation).^103^ Studies have shown that this system is underdeveloped during the initial neonatal period, with a distinct developmental trajectory shown for the classical and alternative complement pathways and differences in lytic activity over the first 24 months of life.^104^ Deficiency in C9 in particular has been associated with hyporesponsiveness to Gram-negative organisms.^105^ Platelets contain complement factors (C1, C1 inhibitor, factor H) and chondroitin sulfate, a glycosaminoglycan that can trigger complement activation.^103^ How the neonatal platelet interacts with the underdeveloped complement system of infancy is as of yet unclear. Platelets themselves can present antigens to adaptive immune cells utilising the major histocompatibility class I mechanism, possessing all the necessary molecular machinery to stimulate a CD8+ T cell response.^106^

Platelets play a key role in the coordination of all components of the innate immune system, producing an initial response in their own right and interacting with myeloid, lymphoid and humoral components of the initial response to infection. While much work has demonstrated that neonates in particular have deficiencies in generating appropriate immune response to infections, enormous gaps remain in the understanding of innate immune system ontogenesis. Further understanding of how platelets interact in this nascent innate immune system, in different contexts and in response to different threats, will be key to understanding the nature of neonatal and paediatric sepsis.

## Platelets and the adaptive immune response in paediatric sepsis

Platelets also shape the adaptive response to infective stimuli. However, the relationships between platelets and the adaptive immune effector cells are less well defined. As discussed above, platelets express surface markers which are key for the differentiation (p-selectin) and activation (CD40L) of DCs.^99,100^

A key step in the formation of an adaptive response is the formation of a germinal centre. This is where DCs presenting an antigen interact with T and B cells possessing a cognate receptor, allowing for clonal expansion and somatic hypermutation of B cells and the generation of long lasting adaptive immunity.^107^ While the formation of both memory B cells and high affinity IgG antibody-producing plasma cells is traditionally thought of as T cell dependent, the rapid presence of high affinity immunoglobins following initial exposure to a pathogen in vivo argues against this.^108^ Platelets have been hypothesised to act during this first phase of the development of humoral immunity. A number of features of platelet physiology strongly support this hypothesis. They are plentiful in circulation, and have been shown to interact with both pathogens and innate immune effectors including DCs. A study conducted in a C57BL/6 mouse model demonstrated that CD40L-expressing platelets were key to generating a robust germinal centre response provided there was also functional CD4+ T cells present.^109^

There is an altered CD4+ T cell phenotype in the neonatal period and infancy, with an initial skew towards a T helper 2(Th2) anti-inflammatory phenotype, which slowly falls during childhood and is replaced by a T helper 1 (Th1) pro-inflammatory response. This has mostly been investigated in the context of the early life development of allergic disease; however, this “tolerogenic” phenotype has also been postulated to contribute to the high incidence of sepsis in the neonatal population.^110^ While the specific role of neonatal platelets in the formation of early life germinal matrix and in T helper cell maturation has not been assessed formally, the hyporeactive phenotype of neonatal platelets may lend itself to a Th2 skewed adaptive immune response as a result of the absence of Th1 inducing signals.^111^ This is an important consideration, as a large number of septic neonates and infants will receive (adult derived) platelet transfusions due to sepsis-related thrombocytopenia. When taken in the context of the PlaNet-2 trial, which demonstrated a liberal platelet transfusion threshold resulted in worse outcomes over a restrictive threshold, consideration should be given to the generation of a pro-inflammatory Th1 phenotype secondary to an exogenous mature platelet infusion.^112^ Adult platelets have been shown to be powerful regulators of both a Th1 and T helper 17 (Th17) response in the context of atherosclerosis, but less impact on Th2 type responses.^113^

Research conducted in a paediatric intensive care setting suggests that typically a more liberal transfusion threshold is applied, often in infant (<1 year old cohort) and that children who received platelet transfusions were more likely to suffer multiorgan failure.^114^ While a cautious interpretation of this retrospective study is warranted, especially as thrombocytopenia in itself is a predictor of a worse outcome, careful consideration of the clinical requirement for platelet transfusion in this vulnerable population may be justified until stronger prospective evidence is gathered.^14^

Given the frequency of use of platelet transfusions not only in neonatal but also in early childhood septic illness, further scientific and clinical investigations are warranted into how platelets and T and B cells interact in the context of paediatric septic illness in the formation of germinal centres.

## Platelet-derived EVs and the platelet proteome/secretome in sepsis

EVs are endogenously produced nanoparticles released by a range of cell types. These EVs allow communication by packaging cellular contents (nucleic acids, proteins, lipid mediators) and allowing them to be taken up by distant cells.^115^ Platelet-derived EVs (EVs) are among the highest concentration in peripheral circulation.^116^ EVs contain many of the immune mediators, including cytokines, chemokines, complement factors and surface receptors such CD40L.^117–120^ They may act as important “warning” signals in sepsis, being released from a site of localised infection and priming a systemic immune response.^117^ Two major subsets of EVs exist, microparticles and exosomes. Microparticles (alternatively referred to as ectosomes/PMPs) are larger (0.1–1 µM) and are generated by major cytoskeletal rearrangements resulting in “shedding”. Exosomes are smaller particles (30–90 nM) and are released from platelets in substantial numbers as part of their “releasate”.^121,122^ Both vesicular categories have been associated with immune functions. PMPs from healthy volunteers have shown to be immunologically active, downregulating NK cell function, DC function and macrophage activation.^123,124^ Platelet-derived exosomes have been shown to contribute to myocardial dysfunction and NET formation.^125,126^ Interestingly, exosome production by platelets was tightly associated with IκB kinase (IKK) in the context of sepsis linking production of these EVs to PRR signalling.^126^

A recent paper showed that neonatal EVs from umbilical cord blood are different in size, concentration and in their protein content from adult EVs, with an increased expression of prothrombotic proteins and a lower expression of immunoglobulins.^127^ This supports previous work from our group examining size and concentration of neonatal EVs and how this alters in the first 3 days in premature infants.^128^

In addition to EVs, degranulation is associated with the release of a range of proteins termed the platelet releasate. Recent work has shown changes in platelet proteome and transcriptome in septic and non-septic patients and animal models.^129,130^ Expression of heparinase increases in the platelets of septic patients as opposed to healthy, matched controls. Increasing heparinase expression is associated with worse clinical outcome, possibly due to loss of the protective vascular glycocalyx leading to oedema and vascular injury.^131^ Studies using the platelet releasate from healthy volunteers induced an anti-inflammatory phenotype in DCs similar to that elucidated by PMPs.^132^ Given the changes in the proteome and transcriptome in the context of sepsis, it would be interesting to examine whether this represents a context-dependent effect or is a general feature of the platelet releasate.

## Conclusions

There is an increasing focus on platelets as key regulators of the immune system. The infant immune system is characterised by a hyporesponsive, tolerogenic phenotype that changes over time during the paediatric period to a pro-inflammatory phenotype in adulthood. The exact ontogeny of individual components of the immune system, and how they interact with platelets to drive the response to infectious agents, is of particular importance in understanding paediatric sepsis and a range of other important inflammatory conditions. Understanding how platelets act as components of the immune system may be key in developing a common definition of sepsis across age groups.

The clinical assumption that paediatric platelets and transfused adult platelets possess a similar phenotype is likely flawed, and underestimates the impact that the more reactive, adult platelet may have on the infant immune response. Thrombocytopenia is a common occurrence in sepsis in children and neonates, with trial data suggesting that increased platelet transfusions result in worse outcomes in neonates.^112^ This is postulated to be due to a “developmental haemostatic mismatch risk”, where adult platelets compromise the delicate homoeostasis of neonatal bleeding.^133^ Platelet transfusions with relatively hyperactive platelets and immunologically active EVs may also pose a “developmental immunological mismatch risk” which, in the presence of an inflammatory stimulus like sepsis, may inadvertently impair outcomes. High quality, prospectively collected data on how best to support neonates, infants and children with low platelet counts is urgently required.

EVs are demonstrably different between neonates and adults. Understanding the normal range of EV release, EV content in sepsis versus health and the roles of EVs versus platelets themselves may represent an important source of sepsis biomarker discovery in the future.
